# The future of patient safety in Ghana: challenges and opportunities

**DOI:** 10.3389/frhs.2025.1581468

**Published:** 2025-07-08

**Authors:** Augustine Kumah

**Affiliations:** Quality Department, The Bank Hospital, Accra, Ghana

**Keywords:** adverse events, patient safety, quality care, quality improvement, Ghana

## Abstract

Patient safety is a critical aspect of healthcare that ensures the reduction of preventable harm to patients during the provision of care. Patient safety has recently gained increasing attention in Ghana, but significant challenges remain. These include inadequate healthcare infrastructure, workforce shortages, medication errors, hospital-acquired infections, and poor reporting and monitoring systems. Moreover, limited public awareness and weak regulatory enforcement exacerbate patient safety risks. However, Ghana also has several opportunities to improve patient safety. Adopting digital health solutions, investing in healthcare worker training, strengthening regulatory frameworks, and community engagement present promising pathways for progress. The government's commitment to Universal Health Coverage (UHC) and recent healthcare policy reforms provide an enabling environment to enhance patient safety initiatives. This article explores the future of patient safety in Ghana by examining key challenges and potential opportunities. It provides an in-depth analysis of patient safety, discusses barriers to improvement, and highlights strategies to enhance safety outcomes. The article also examines global best practices that can be adapted to the Ghanaian healthcare system. Improving patient safety in Ghana requires a multi-stakeholder approach, integrating policy reform, technology, workforce development, and public awareness campaigns. By leveraging existing opportunities and addressing challenges, Ghana can move toward a safer, more effective healthcare system that protects patients from preventable harm.

## Introduction

Ghana's healthcare system is a decentralized, mixed delivery model comprising public, private, and faith-based providers, coordinated primarily through the Ministry of Health (MoH) and implemented by the Ghana Health Service (GHS) (1). The country operates a three-tiered system—primary, secondary, and tertiary levels—anchored by a network of community-based health planning and services (CHPS) compounds, district hospitals, and teaching hospitals (1, 2). The National Health Insurance Scheme (NHIS), introduced in 2003, aims to improve financial access to healthcare, though challenges with coverage, equity, and service quality remain (1–3). Despite notable progress in expanding access to care, the sector is constrained by limited infrastructure, inadequate staffing, and inequities in resource distribution—particularly between urban and rural areas (1, 2, 4, 5). These systemic weaknesses have direct implications for patient safety, particularly in settings where overcrowding, staff burnout, and poor infection control are prevalent (3). As global attention intensifies on reducing preventable harm in healthcare, understanding patient safety within the Ghanaian context is critical. The country's healthcare challenges—ranging from medication errors to underreporting of adverse events—underscore the urgent need for comprehensive safety frameworks. Against this backdrop, patient safety has emerged as a national priority, though significant gaps in implementation and awareness persist (2, 3).

Patient safety is a fundamental principle in healthcare aimed at preventing errors, reducing harm, and ensuring high-quality care delivery. The World Health Organization (WHO) defines patient safety as the “absence of preventable harm to a patient during the process of health care and the reduction of risk of unnecessary harm associated with health care to an acceptable minimum” (6, 7). It encompasses various strategies, policies, and systems designed to improve the safety of healthcare delivery and enhance patient outcomes (7).

Ensuring patient safety is essential in reducing medical errors, which are among the leading causes of morbidity and mortality worldwide. Studies have shown that adverse events occur in approximately 10% of hospital admissions, a significant proportion being preventable (8–10). These errors range from medication mistakes and surgical complications to healthcare-associated infections (HAIs) and diagnostic inaccuracies (10, 11). By implementing robust patient safety measures, healthcare organizations can mitigate risks, improve efficiency, and enhance patient trust in the healthcare system.

Despite progress in patient safety initiatives, several challenges persist. These include inadequate staffing, burnout among healthcare workers, poor communication, and resistance to change (6, 7, 11). Additionally, disparities in healthcare access and resource limitations in low- and middle-income countries hinder the implementation of effective safety measures (6, 7). This is a perspective article which explores the major themes affecting patient safety in Ghana.

## The state of patient safety in Ghana

Patient safety has become a global healthcare priority, with international organizations such as the WHO and the Institute for Healthcare Improvement (IHI) emphasizing its significance (7, 12). Patient safety has gradually gained recognition in Ghana, but significant challenges persist. A GHS study revealed that medical errors, poor infection control, and misdiagnoses are common in healthcare settings (2). Although Ghana has made strides in healthcare access, safety standards have not kept pace with the increasing demand for services (2).

One of the most pressing concerns is hospital-acquired infections (HAIs), which continue to be a major cause of morbidity and mortality (11). Poor hand hygiene, inadequate sanitation, and overcrowded hospitals contribute to HAIs. In addition, medication safety remains a critical challenge, with reports of prescription errors, counterfeit drugs, and self-medication posing serious risks to patients (3, 11, 13).

## Methods

This perspective article employed a non-systematic search and thematic content analytical approach to explore the state of patient safety in Ghana, synthesizing available literature, policy documents, and secondary data sources. The objective was to critically examine the trends, challenges, and opportunities for improving patient safety within the Ghanaian healthcare context, drawing on both national and international best practices.

A non-systematic search of the literature published between 2015 and 2024 was conducted across multiple databases, including PubMed, Scopus, Web of Science, Google Scholar, and the WHO Institutional Repository. The search strategy used key terms such as “patient safety”, “adverse events”, “hospital-acquired infections”, “medication errors”, “quality improvement”, and “Ghana”. Boolean operators (AND, OR) were employed to refine search outputs. In addition to international literature, key national reports such as the Ghana National Healthcare Quality Strategy (2016–2026), Ghana Health Service Annual Reports, and Ministry of Health policy briefs were reviewed to provide a locally relevant and updated perspective. Comparative insights were drawn from global patient safety strategies, such as the NHS Patient Safety Strategy (UK), NSQHS Standards (Australia), and accreditation frameworks from Canada and the United States.

The interpretation of data and formulation of recommendations were informed by the author's professional experience in healthcare quality improvement and public health, incorporating practical insights into the feasibility of proposed interventions. Where necessary, the article triangulates empirical findings with expert perspectives and policy analysis to ensure relevance and contextual accuracy.

No data was collected, and ethical approval was not required. All data used are publicly available and appropriately cited.

## Results

Key thematic areas were identified through content analysis of patient safety challenges in Ghana's healthcare system, structured around key thematic areas: adverse events and hospital-acquired infections (HAIs), medical errors and diagnostic failures, systemic healthcare weaknesses, and medication safety. These themes illustrate systemic inefficiencies that hinder the delivery of safe and quality care, while also highlighting avenues for targeted reforms.

Hospital-acquired infections remain a serious burden. A 2016 point-prevalence study found an 8.2% HAI rate across ten hospitals, with surgical site infections (32.6%), bloodstream infections (19.5%), and urinary tract infections (18.5%) being most prevalent (14). Recent data from 2024 estimates the economic burden of HAIs at $1.57 billion annually—about 1.98% of Ghana's GDP (15). Alarmingly, 45% of healthcare facilities lack access to clean water, significantly increasing infection risk and undermining infection prevention and control (IPC) practices (15, 16).

Medical errors, including diagnostic and medication-related mistakes, are widespread. Errors in dosage, frequency, and drug-treatment alignment are common, often stemming from poor documentation practices, inadequate verification, and systemic oversight failures (13, 17, 18). Diagnostic inaccuracies—such as incompatible blood transfusions and misidentified genotypes—frequently lead to avoidable morbidity and mortality. Experts emphasize the importance of systemic reforms, including mortality audits, autopsies, and case reviews, to reduce such events (13, 19, 20).

Healthcare system weaknesses further exacerbate patient safety issues. Ghana faces critical shortages and imbalances in the distribution of the healthcare workforce (4). The disparity is especially pronounced in rural areas, where understaffing contributes to overburdened health workers and reduced quality of care (4). Infrastructure challenges persist as well, with only 5% of primary health facilities equipped with complete basic examination tools (3). Financial constraints are evident, with only 6.9% of the 2024 national budget allocated to health, far below the 15% Abuja Declaration target (21). Moreover, 71% of the health budget is spent on salaries, leaving limited resources for service improvement (21).

Medication errors are notably high. A 2024 pediatric study showed that 68.4% of children experienced at least one medication error at home, mainly involving wrong timing (45.1%) and dosing frequency (21.6%) (22). Hospital-based studies report error rates of 27.2% and 18.2%, the latter with nearly half resulting in adverse events (17). These are largely attributed to inadequate training, the absence of standardized guidelines, and a high workload (17, 22).

## Discussion

### Challenges to patient safety in Ghana

#### Healthcare infrastructure deficiencies

Ghana's healthcare system faces significant resource limitations, particularly in rural areas. One of the major threats to patient safety in Ghana is the inadequate healthcare infrastructure. Many hospitals and clinics, especially in rural areas, lack essential medical equipment, clean water, and proper sanitation, increasing the risk of infections and poor patient outcomes (4). Overcrowding in hospitals and limited resources make it difficult for healthcare workers to provide safe and effective care.

#### Workforce shortages and training gaps

The shortage of skilled healthcare professionals further exacerbates patient safety concerns. According to the WHO, Ghana's doctor-to-patient ratio remains critically low at approximately 1:8,000, far below the recommended 1:1,000 (6). Many hospitals operate with insufficient nursing staff, leading to burnout, fatigue, and an increased likelihood of medical errors (4). Additionally, ongoing professional development programs focusing on patient safety are limited, leaving many healthcare workers without updated knowledge of best practices (3, 4, 11).

The concept of healthcare quality improvement (QI) is becoming increasingly central to the global push for better health systems. QI is a systematic, data-driven approach to improving healthcare processes and enhancing care quality, safety, efficiency, and patient-centered care (23). Unfortunately, Ghana's healthcare training programs predominantly focus on clinical and theoretical knowledge, with little emphasis on system-based practices such as quality improvement (3, 23, 24). This gap in training means that many health professionals enter the workforce without the skills necessary to critically evaluate and improve healthcare delivery processes. As a result, the healthcare system struggles with inefficiencies that negatively impact patient outcomes, access to care, and overall system sustainability (11).

#### Medication errors and unsafe prescribing practices

Medication errors are another major issue affecting patient safety in Ghana. Prescription errors, lack of patient education on drug usage, and counterfeit medications contribute to adverse drug reactions (25, 26). Ghana's Food and Drugs Authority (FDA) has tried to regulate the pharmaceutical sector, but enforcement challenges persist, leading to the continued circulation of substandard medications (1, 2).

#### Infection prevention and control (IPC) issues

Despite efforts to improve infection control, many health facilities in Ghana still struggle with implementing robust IPC measures (2, 3, 24). HAIs remain a significant concern due to shortages PPEs and inadequate infection control measures (24). Many hospitals struggle with maintaining proper hygiene practices, including handwashing, sterilization of medical equipment, and safe waste disposal (3, 24). The COVID-19 pandemic exposed weaknesses in Ghana's infection prevention systems, highlighting the urgent need for sustainable IPC interventions (27).

#### Weak health information and reporting systems

A significant barrier to improving patient safety is the absence of a robust reporting system for adverse events. Medication administration errors (MAE), adverse events, and incident reporting are critical to patient safety and healthcare quality improvement (11, 25, 26). Effective MAE reporting systems encourage healthcare professionals to report errors without fear of punishment, fostering a culture of transparency and continuous learning (25). However, several factors influence healthcare professionals' willingness and ability to report MAEs (25). Fear of punitive measures discourages healthcare workers from reporting medical errors, limiting opportunities for learning and systemic improvements (13, 25, 28). Without reliable data on patient safety incidents, policymakers face challenges in identifying key areas for intervention.

#### Lack of patient safety culture and reporting mechanisms

A significant barrier to improving patient safety is the lack of a strong reporting culture for adverse events (25). Healthcare workers fear punitive actions when they report medical errors, leading to underreporting and missed opportunities for system-wide improvements (23). Establishing a non-punitive, learning-oriented reporting system is essential for patient safety advancement (29).

## Opportunities for improving patient safety in Ghana

Patient safety is a fundamental aspect of healthcare delivery, ensuring patients receive care with minimal risk of harm. Despite the numerous challenges affecting patient safety in Ghana, there are significant opportunities for improvement. With strong policy commitments, technological advancements, and strategic investments in healthcare infrastructure, Ghana can enhance patient safety outcomes. Key areas of opportunity include strengthening healthcare infrastructure, improving workforce capacity, leveraging digital health technologies, enhancing medication safety, fostering a culture of transparency, and engaging communities in patient safety efforts.

## Strengthening healthcare infrastructure

A well-equipped healthcare system is essential for patient safety. Many health facilities in Ghana, particularly in rural areas, lack basic medical equipment, clean water, and proper sanitation, which increases the risk of infections and poor clinical outcomes (3, 24). To address this, increased investment in healthcare infrastructure is needed. Public-private partnerships (PPPs) can also be vital in expanding and modernizing healthcare facilities. The Ghanaian government has committed to improving healthcare infrastructure through the Agenda 111 initiative to build 111 new hospitals nationwide. Ghana's Agenda 111 initiative aims to provide every district and region in Ghana with access to quality healthcare. The project, spearheaded by the Ministry of Health, is a key part of the government's vision of universal health coverage. It focuses on improving the health of all people in Ghana. The completion of these new hospitals, coupled with increased funding for essential medical supplies and equipment, will ensure safer healthcare delivery.

Upgrading Rural Healthcare Facilities: Many rural health centers lack essential services such as emergency care and advanced diagnostic tools. Expanding telemedicine services and equipping health posts with modern technology can help bridge these gaps (30). Providing a reliable electricity and water supply in these facilities is critical for ensuring safe and hygienic care.

## Enhancing workforce capacity and training

A well-trained healthcare workforce is crucial for ensuring patient safety. In Ghana, the shortage of healthcare professionals and the lack of continuous training programs contribute to patient safety risks (4). Addressing this gap can significantly improve healthcare outcomes. The government must increase efforts to train and retain healthcare professionals, especially in underserved areas. Incentives such as housing, salary increments, and professional development opportunities can encourage doctors and nurses to work in rural communities (4).

Training in patient safety, infection control, and evidence-based practices should be mandatory for all healthcare workers. Simulation-based training can help doctors and nurses practice emergency response protocols in a controlled environment, reducing errors in real-life scenarios (3, 31). Improving Nurse-to-Patient Ratios: Overburdened nurses are more likely to make errors that compromise patient safety. Increasing the recruitment of nurses and implementing shift rotations can help reduce fatigue and enhance patient care quality (25).

## Leveraging digital health and technology

Integrating digital health technologies presents a significant opportunity to improve patient safety in Ghana. Electronic health records (EHRs), telemedicine, and artificial intelligence (AI) can enhance healthcare efficiency and reduce medical errors (11, 31). Many hospitals in Ghana still rely on paper-based patient records, increasing the risk of documentation errors and miscommunication (3, 24). EHRs can improve data accuracy, facilitate coordination among healthcare providers, and enhance patient safety. The government should invest in a nationwide EHR system to ensure seamless data sharing across health facilities.

Telemedicine can help address geographical barriers to healthcare access, especially in rural areas (30). Virtual consultations reduce unnecessary hospital visits, minimizing the risk of hospital-acquired infections (31). Internet infrastructure improvements in remote areas should accompany the expansion of telemedicine services. AI-powered systems can assist healthcare workers in diagnosing diseases, predicting patient deterioration, and reducing diagnostic errors (11, 26, 32). Implementing AI-driven decision support tools can improve clinical decision-making and patient outcomes.

## Enhancing medication safety and pharmacovigilance

Medication errors remain a significant patient safety issue in Ghana. Implementing robust pharmacovigilance systems and regulatory measures can help prevent adverse drug reactions and counterfeit drug circulation (32, 33). Ghana needs to strengthen drug regulation and surveillance. The Ghana Food and Drugs Authority (FDA) has progressed in regulating medications, but enforcement remains inconsistent (34). Strengthening surveillance systems to track and remove counterfeit drugs from the market can enhance medication safety (35, 36).

Healthcare providers should receive training on medication safety, including appropriate prescribing practices and drug interactions. Hospitals should implement electronic prescribing (e-prescriptions) to reduce the risk of prescription errors (13, 26, 31). Many patients self-medicate due to a lack of awareness about the dangers of improper medication use. Public education campaigns should emphasize the importance of adhering to prescribed treatments and the risks of buying drugs from unauthorized vendors (31).

## Fostering a culture of transparency and patient safety reporting

A strong patient safety culture depends on transparency, learning from errors, and fostering an environment where healthcare workers can report mistakes without fear of punishment (11, 13, 19, 20, 37). Fear of disciplinary action discourages healthcare workers from reporting medical errors, limiting opportunities for system-wide improvements (11, 25). Establishing a confidential, non-punitive reporting system can encourage open discussions on patient safety incidents (29). In addition, hospital administrators and policymakers should prioritize patient safety by implementing clear error prevention and response protocols. Regular safety audits and feedback mechanisms can help track progress and address gaps in healthcare delivery (8, 37, 38). A significant factor in enhancing reporting rates is establishing a “just culture”, emphasising accountability and learning over punitive measures (29, 39). While just culture significantly enhances adverse event reporting, its successful implementation requires robust commitment at all organisational levels.

## Community engagement and patient participation

Patient safety improvements should not be limited to hospitals and healthcare professionals; patients and communities must also be actively involved. Educating patients about their rights can empower them to demand quality care and report safety concerns (40). Public campaigns should focus on hygiene practices, proper medication use, and recognizing medical errors. Hospitals should establish patient feedback systems to identify concerns and improve service delivery. Platforms such as mobile apps and toll-free helplines can enable patients to report safety incidents in real-time (41).

## Learning from global best practices

Improving patient safety is a global priority, with many countries implementing best practices to minimize preventable harm in healthcare settings. Ghana can learn from these experiences by adopting effective policies, leveraging digital health technologies, and fostering a culture of transparency and accountability. Countries such as the United Kingdom, Canada, Australia, and the United States have developed comprehensive patient safety strategies that could be adapted to fit Ghana's healthcare system (7).

One of the key lessons from global best practices is the importance of standardized patient safety protocols. The United Kingdom's National Health Service (NHS) has implemented the National Patient Safety Strategy, which includes guidelines on infection prevention, medication safety, and clinical decision-making (9, 42). Australia's National Safety and Quality Health Service (NSQHS) Standards provide a framework for hospitals to maintain high safety standards (42, 43). Ghana can adopt similar national guidelines (42, 43). Ghana can adopt similar national guidelines, ensuring all healthcare facilities follow standardized patient safety protocols.

Countries like Canada and the United States have established accreditation bodies, such as the Canadian Patient Safety Institute (CPSI) and The Joint Commission (TJC), which certify hospitals based on compliance with safety standards (43). Ghana can strengthen its Health Facilities Regulatory Agency (HeFRA) to enforce hospital accreditation, ensuring adherence to best practices in patient care.

## Conclusion

The future of patient safety in Ghana hinges on its ability to address its existing healthcare challenges while leveraging new opportunities for improvement. While inadequate infrastructure, workforce shortages, medication errors, and poor infection control remain significant barriers, several initiatives offer hope for progress.

Investments in healthcare infrastructure, digital health technologies, and workforce training can significantly enhance patient safety outcomes. Strengthening medication safety regulations, improving infection control practices, and fostering a transparent reporting culture will reduce preventable harm. Additionally, integrating patient-centered approaches through community engagement and health literacy programs will empower citizens to take an active role in their safety. Improving patient safety in Ghana requires a coordinated, multi-stakeholder approach involving the government, healthcare professionals, regulatory bodies, and patients. By addressing systemic gaps and embracing innovation, Ghana can build a resilient healthcare system that prioritizes patient safety and ensures better health.

## Data Availability

The original contributions presented in the study are included in the article/Supplementary Material, further inquiries can be directed to the corresponding author.
